# Development of the First Episode Digital Monitoring mHealth Intervention for People With Early Psychosis: Qualitative Interview Study With Clinicians

**DOI:** 10.2196/41482

**Published:** 2022-11-04

**Authors:** Ana Stefancic, R Tyler Rogers, Sarah Styke, Xiaoyan Xu, Richard Buchsbaum, Ilana Nossel, Leopoldo J Cabassa, T Scott Stroup, David Kimhy

**Affiliations:** 1 Department of Psychiatry Vagelos College of Physicians and Surgeons Columbia University New York, NY United States; 2 Division of Behavioral Health Services and Policy Research New York State Psychiatric Institute New York, NY United States; 3 Department of Biostatistics Mailman School of Public Health Columbia University New York, NY United States; 4 Brown School of Social Work Washington University in St Louis St Louis, MO United States; 5 Department of Psychiatry Icahn School of Medicine New York, NY United States; 6 New York Mental Illness Research Education and Clinical Center The James J Peters Veteran's Affairs Medical Center Bronx, NY United States

**Keywords:** first-episode psychosis, early psychosis, coordinated specialty care, mental health treatment, shared decision-making, mobile health, smartphone apps, qualitative, digital psychiatry, mobile phone

## Abstract

**Background:**

Mobile health (mHealth) technologies have been used extensively in psychosis research. In contrast, their integration into *real-world* clinical care has been limited despite the broad availability of smartphone-based apps targeting mental health care. Most apps developed for treatment of individuals with psychosis have focused primarily on encouraging self-management skills of patients via practicing cognitive behavioral techniques learned during face-to-face clinical sessions (eg, challenging dysfunctional thoughts and relaxation exercises), reminders to engage in health-promoting activities (eg, exercising, sleeping, and socializing), or symptom monitoring. In contrast, few apps have sought to enhance the clinical encounter itself to improve shared decision-making (SDM) and therapeutic relationships with clinicians, which have been linked to positive clinical outcomes.

**Objective:**

This qualitative study sought clinicians’ input to develop First Episode Digital Monitoring (FREEDoM), an app-based mHealth intervention. FREEDoM was designed to improve the quality, quantity, and timeliness of clinical and functional data available to clinicians treating patients experiencing first-episode psychosis (FEP) to enhance their therapeutic relationship and increase SDM.

**Methods:**

Following the app’s initial development, semistructured qualitative interviews were conducted with 11 FEP treatment providers at 3 coordinated specialty care clinics to elicit input on the app’s design, the data report for clinicians, and planned usage procedures. We then generated a summary template and conducted matrix analysis to systematically categorize suggested adaptations to the evidence-based intervention using dimensions of the Framework for Reporting Adaptations and Modifications‐Enhanced (FRAME) and documented the rationale for adopting or rejecting suggestions.

**Results:**

The clinicians provided 31 suggestions (18 adopted and 13 rejected). Suggestions to add or refine the content were most common (eg, adding questions in the app). Adaptations to context were most often related to plans for implementing the intervention, how the reported data were displayed to clinicians, and with whom the reports were shared. Reasons for suggestions primarily included factors related to health narratives and priorities of the patients (eg, focus on the functional impact of symptoms vs their severity), providers’ clinical judgment (eg, need for clinically relevant information), and organizations’ mission and culture. Reasons for rejecting suggestions included requests for data and procedures beyond the intervention’s scope, concerns regarding dilution of the intervention’s core components, and concerns about increasing patient burden while using the app.

**Conclusions:**

FREEDoM focuses on a novel target for the deployment of mHealth technologies in the treatment of FEP patients—the enhancement of SDM and improvement of therapeutic relationships. This study illustrates the use of the FRAME, along with methods and tools for rapid qualitative analysis, to systematically track adaptations to the app as part of its development process. Such adaptations may contribute to enhanced acceptance of the intervention by clinicians and a higher likelihood of integration into clinical care.

**Trial Registration:**

ClinicalTrials.gov NCT04248517; https://tinyurl.com/tjuyxvv6

## Introduction

### Early Intervention for Psychosis and Measurement-Based Care

Early treatment experiences of individuals diagnosed with schizophrenia can have enduring effects on their attitudes toward treatment, potentially altering the course of illness and affecting long-term outcomes [1,2]. Consequently, first-episode psychosis (FEP) is a critical period for optimizing treatment to enhance treatment satisfaction and adherence [3]. Specifically, psychotropic medications are critical core components of early intervention strategies. However, evidence suggests that a significant gap exists between the optimal use of medications and how they are used in real-world practice [4], with many patients receiving higher than recommended dosages of antipsychotic medications, as well as additional psychotropic medications. These practices often result in troubling symptoms and side effects, lower satisfaction with treatment, poorer therapeutic relationship and treatment engagement, and increased rates of discontinuation of treatment [1,4].

One widely promoted approach to improve treatment outcomes is measurement-based care (MBC), which is defined as the systematic evaluation of patient conditions before or during an encounter to inform treatment [5,6]. Typically, MBC relies on the patients’ recollection of their clinical status over several days or weeks. However, such retrospective assessments are problematic because they are vulnerable to the influence of memory difficulties, cognitive biases, and reframing [7-9]. These issues are particularly salient among individuals with schizophrenia, given the substantial episodic memory deficits documented in this population [10,11]. In addition, medication management sessions by psychiatrists and other prescribing clinicians typically last <30 minutes, making it difficult for providers to obtain a comprehensive view of the clinical status of patients and develop rapport. The latter is particularly pertinent for patients with FEP, about whom psychiatric care providers may have shorter treatment histories, resulting in less familiarity. Overall, these limitations may contribute to lower treatment satisfaction and adherence, poorer therapeutic relationship, and poorer clinical outcomes.

A promising strategy to overcome many of these challenges is the use of mobile health (mHealth) technologies. Extensive evidence from psychosis research studies using smartphones indicates high feasibility and validity of real-time collection of clinical information on daily experiences among individuals with psychosis, including symptoms, side effects, mood and affective processing, social activities and context, sleep, and functioning [9,12-15]. Using apps and methodologies, such as experience sampling method (ESM) that present patients with brief assessments that are more frequent and richer in detail and occur during the course of “real-world” functioning, mHealth technologies can provide a more granular and complete picture of clinical status and functioning of patients upon which more effective clinical decisions and pharmacological management can be made [9,13,16,17]. Specifically, mHealth technologies can capture changes in clinical variables across time and social contexts, potentially allowing providers to better tailor interventions. Furthermore, the “real-world” characterization of experiences of patients via mHealth technologies may also enhance shared decision-making (SDM) and therapeutic relationship by providing both clinicians and patients with more accurate clinical data that are more directly related to experiences of patients, potentially allowing for more informed joint treatment decisions.

### mHealth Applications in Psychosis Treatment

To date, most apps developed for and used in the treatment of individuals with psychosis, including FOCUS [18-20], CORE [21], Actissist [22], and Acceptance and Commitment Therapy in Daily Life [23], have focused largely on supplementing face-to-face clinical encounters [16,17,24-28]. Most apps have been designed primarily to facilitate patients’ self-management of symptoms and recovery by offering psychoeducation, guidelines for practicing of cognitive and behavioral coping strategies (eg, reassessment of dysfunctional beliefs and relaxation exercises), or other skills taught during clinical sessions. Other apps have focused on monitoring symptoms and signs of clinical deterioration or enhancing social functioning [29-32]. In contrast, few apps have sought to enhance the clinical encounter itself. Specifically, to date, no app has aimed to enhance SDM and therapeutic relationships within FEP treatment. SDM has been shown to be a key element contributing to positive clinical outcomes [33,34]. Previous reports have demonstrated that SDM has a positive impact on patient satisfaction, adherence to treatment, quality of life, and empowerment, including among patients with serious mental illness [35-38]. Consistent with this view, Zielesak et al [39] pointed out that there remains a significant gap in understanding clinician needs for information in mental health care decision-making, as well as ways to better integrate apps into routine clinical care and provider workflow. Furthermore, providers’ lack of engagement with, or buy-in for, patient-reported health data have been noted as a critical barrier to its use in health care generally, making it a priority to elicit adaptations that may facilitate uptake from the provider perspective [40].

To address these gaps in the literature, we sought to develop an mHealth intervention that provides psychiatric care providers with clinically relevant and time-sensitive information that would enhance MBC, better inform decisions regarding treatment and medication management, and improve therapeutic relationships and SDM. Prior research has demonstrated the benefits of soliciting stakeholder input when developing and refining mHealth apps, including for individuals with schizophrenia [19] and early psychosis [22,31,41]. For example, Ben-Zeev et al [19] used a multistage, multistakeholder input and feedback approach combining survey and qualitative methods to develop the FOCUS app that supports self-management for people with schizophrenia. Similarly, within an early intervention service for psychosis, McClelland and Fitzgerald [41] conducted a staged series of focus groups with patients and clinicians to develop an app that helped patients track their mood and activities, receive reminders and messages, and seek external support.

In this study, we described the systematic process of soliciting inputs from clinician stakeholders to develop and adapt an app as part of a pilot study examining the implementation of a community-based FEP mHealth intervention for adolescents and young adults. As our app focuses on a novel clinical target, the information available to clinicians, and its use in SDM, the views and input of clinicians were critical for elucidating this target. Adaptations may entail changes to interventions, or to implementation strategies, that produce better alignment with factors such as the needs, resources, and cultures of target settings and populations [42]. Specifically, such input may lead to adaptations in multiple aspects of an intervention, including content, frequency, and timing, which may then improve intervention fit (eg, appropriateness), feasibility (eg, successful delivery), acceptability (eg, satisfactoriness), and effectiveness, given a particular practice setting and population served or higher-level contextual factors such as local policies [43]. Changes to implementation strategies can include adding intervention training or modifying workflows, as these focus on methods and activities that seek to maximize the extent to which an intervention is adopted, used, and sustained within routine practice [44]. Overall, adaptations may address several considerations, including clinical judgment, stakeholder preferences, and perception of the intervention, as well as factors associated with the entity or setting (eg, clinic) within which the intervention is embedded, such as an organization’s access to resources, social context, or mission. Finally, adaptations can also be responsive to the wider sociopolitical context, such as social norms or mores, and funding policies.

In addition to the practical value of obtaining stakeholder input for intervention design, there are increasing calls for the development, tracking, and reporting of processes and findings regarding adaptations to interventions and implementation strategies as part of efforts to disseminate methods, tools, and resources that promote rapid and iterative applications of implementation science and translational research [45]. One such tool is the Framework for Reporting Adaptations and Modifications‐Enhanced (FRAME) [46,47]. It facilitates the ability of researchers and providers to capture a range of information relevant to adaptation decision-making processes and to catalog ways in which a practice has changed from a previously established iteration or protocol. The FRAME allows for systematic classification of intervention adaptations by guiding researchers and providers to address key questions such as (1) when adaptations are made; (2) who participated in the decision-making process; (3) specifically, what was modified or adapted and to which aspect of the intervention does it relate (eg, context and content); (4) the reasons why an adaptation was made; (5) the goal of the adaptation (eg, increase reach or engagement); and (6) whether the adaptation is consistent with intervention fidelity or an intervention’s core principles.

## Methods

### Context and Setting

This qualitative study was conducted as part of a pilot randomized controlled trial (RCT) to evaluate the feasibility and acceptability of using First Episode Digital Monitoring (FREEDoM), a novel mHealth app designed to enhance MBC and SDM, as well as improve patient satisfaction with pharmacotherapy regimens at 3 clinics delivering coordinated specialty care (CSC) [48,49] for patients with FEP (ClinicalTrials.gov NCT04248517). The clinics, all affiliated with OnTrackNY, provide treatment to adolescents and young adults (aged 16-30 years) experiencing nonaffective FEP [44]. OnTrackNY originated as part of the National Institute of Mental Health Recovery After an Initial Schizophrenia Episode Implementation and Evaluation Study. The CSC programs use an evidence-based, multidisciplinary, and team-based approach that offers pharmacotherapy, psychotherapy, supported employment and education, and peer support and emphasizes an SDM approach to treatment [50]. Semistructured qualitative interviews were conducted with CSC program staff (eg, psychiatric care providers and primary therapists) before initiating the RCT at each site to elicit provider perspectives on the proposed intervention and plans for implementation. Researchers used provider feedback from structured interviews and the FRAME to identify, catalog, track, and implement adaptations to increase the potential feasibility and acceptability of the RCT intervention and protocol.

### FREEDoM—a Novel mHealth Intervention

The FREEDoM mHealth intervention project involved patients completing 3-day ESM-based assessments once per month immediately before their appointment with their psychiatric care provider of the CSC program. The goal of the intervention was to provide timely, accurate, and granular information about clinical status; improve communication about pharmacotherapy between patients and clinicians; enhance SDM; and improve patient treatment satisfaction.

During the 3-day assessment, the mHealth app delivered notifications to the participants’ smartphones 10 times a day at random times between 10 AM and 10 PM to complete brief questionnaires. Participants had 15 minutes to begin responding to questions presented on the smartphone’s screen. The questions asked during each sampling assessment varied based on the time of day and a system of branching logic within each set of questions. The first daily questionnaire included questions about sleep and medications taken the previous day. The middle 8 questionnaires asked about psychiatric symptoms, medication side effects, mood, substance use, social activities, and context, as well as activities and difficulties functioning. The final questionnaire each day asked about side effects that are less transient (eg, constipation and sexual side effects), as well as global functioning. Each questionnaire took 3 to 5 minutes to complete. Following the 3-day ESM assessment, the clinician received a 1-page succinct report summarizing key clinical variables characterizing the current status and functioning of the patient, along with changes from the previous month and the start of the study that could be reviewed and discussed with the patient in the upcoming session. Clinicians were encouraged to share the reports with their patients during clinical sessions and use them as a basis for discussions on clinical status, treatment goals, clinical progress, and SDM.

### Sample

A purposive sampling approach was used to identify staff members at each CSC site whose primary role was to provide clinical care to patients. Team leaders served as initial key informants at each site and nominated a psychiatric care provider (either a physician or nurse practitioner) and other clinical staff members, whom they believed would contribute feedback relevant to the proposed intervention and implementation plan, for study participation. All staff members identified for the interviews provided informed consent and participated in the study.

### Data Collection

The initial development of the questions and inquiry items included in the FREEDoM app was completed by DK and TSS, with the team members providing additional edits. Next, the CSC providers completed individual semistructured interviews lasting approximately 1 hour each. Interviews were conducted by 2 senior MD or PhD clinician researchers (TSS and DK) who were trained and supported by 2 experts in qualitative methods and implementation science (LJC and AS). The first 2 interviews were conducted in-person before the COVID-19 pandemic restrictions, and subsequent interviews were conducted via videoconferencing (eg, via Zoom) owing to social distancing mandates. Interview guides (Multimedia Appendix 1), which were developed collaboratively by the research team, were framed to inquire about providers’ perspectives on study procedures related to implementation, recruitment, and retention, as well as feedback on the content and structure of both the FREEDoM mHealth app used to deliver the proposed intervention and the report delivered to clinicians. During the interviews, providers were shown screenshots of the mHealth app and a draft of the 1-page clinical report for feedback. The interviews were audio-recorded, transcribed verbatim, reviewed for accuracy, and deidentified.

### Pragmatic Data Analysis Procedures

**Figure 1:**
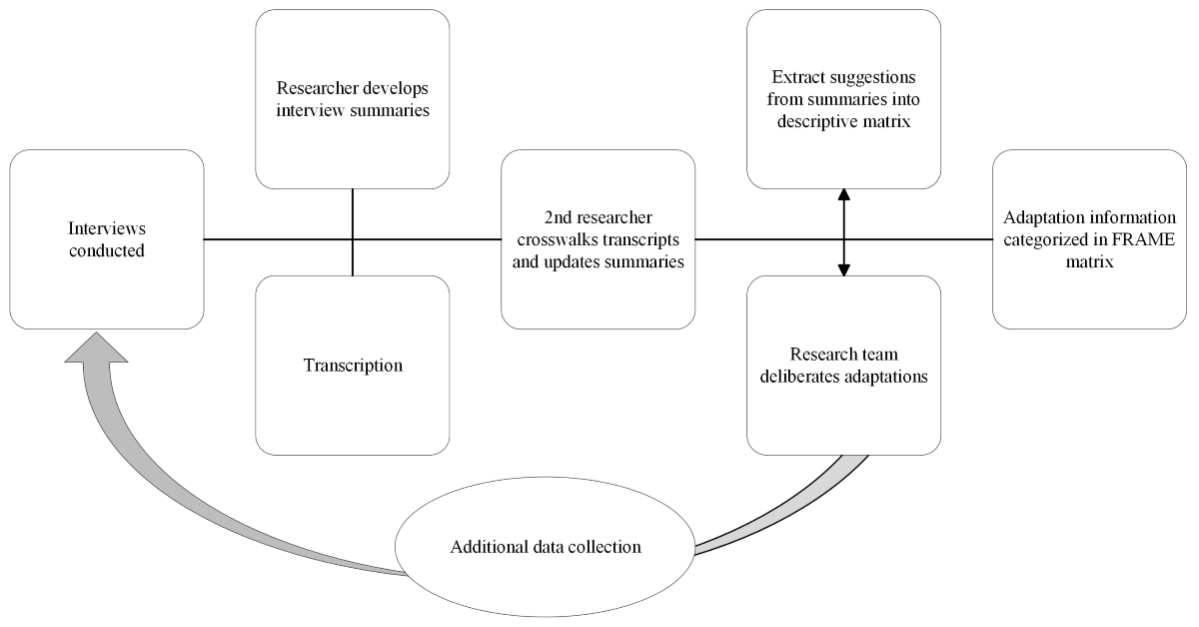
Pragmatic analysis for rapid qualitative research. FRAME: Framework for Reporting Adaptations and Modifications-Enhanced.

Data analysis and deliberation of adaptations were performed in tandem with data collection (Figure 1). Data were analyzed using a summary template and matrix analysis approach to categorize suggested adaptations using key dimensions of the FRAME. Matrix analysis is a rigorous but pragmatic method for rapidly extracting and reducing qualitative data, allowing researchers to systematically synthesize and catalog content into a template of key topics [51-53].

Following the semistructured interviews, one author (RTR) developed draft interview summaries of each transcript, extracting interview content based on key interview topics. These summaries were then edited by a senior author (AS) with expertise in qualitative analysis to ensure that all information pertinent to potential adaptations from each transcript was captured in the summary. Summaries included providers’ assessment of procedures or content (eg, endorsed or had concerns) and systematically outlined each suggested adaptation along with illustrative quotes.

Next, brief descriptions of the suggested adaptations and relevant contextual information from the summaries were entered into a descriptive adaptation matrix (Table 1). The adaptation matrix was a Microsoft Excel table template with column headings representing information that would be needed to classify adaptations along FRAME domains (the adaptation suggested, supporting rationale or contextual information, whether adaptations would vary by study site, and key quotes) and rows outlining potential adaptations organized by project components (eg, “project implementation issues,” “app-related,” and “report-related”) with specific subtopics (eg, “mobile phone and data plan reimbursement” was a subtopic of “project implementation issues”). During this charting process, the authors met every other week to discuss the suggested adaptations and deliberate making changes. Decisions on whether to implement a suggested adaptation were documented by 1 author (RTR) in the adaptation matrix along with a brief description of why the adaptation was incorporated.

**Table 1:** A sample descriptive adaptation matrix mobile health First Episode Digital Monitoring.

Domain		Stakeholder input and feedback	Suggestion for adapted practice	Varies by site	Relevant stakeholder quote	Adaptation implemented
**App-related**						
	Questions or content	Current app content is fine, but weight gain should be added as a side effect	Add weight gain to the app questions	No	“Would you consider adding weight gain to the list of side-effects? Because that’s been something that has been brought up by some participants in the past and the prescriber really tries to, to work with them on that.”	Yes
	Pinging and frequency	10 pings a day may be too much	Reduce pings per day	N/A^a^	“I’m just curious if like if you’re in college, you’re in high school; like how realistic is it that you’re going to be...? I don’t know what’s the frequency...”	No
**Report**						
	Layout or design	Inclusion of daily averages is useful	Include daily averages in granular graph	No	“I like having the average for the day.”	Yes
	Access to report	It would be helpful for participants to receive a report of their answers within the app itself	Consider providing report directly to participant	No	“Just wondering, like are, are participants able to get like information like this?... like maybe like since it’s an app like they’re able to like see what they said and like past months.”	No, consider for future iteration
	Content	Caffeine should be included owing to its effects on sleep	Include caffeine as a substance	No	“[Caffeine and other substances] are relevant for sleep.”	Yes

^a^N/A: not applicable.

After all the suggested adaptations were entered into the descriptive adaptation matrix, each adaptation was further classified along FRAME domains that were applicable to tracking planned adaptations before implementation for interventions without established fidelity standards (what was modified at what level of delivery, type of contextual adaptation, nature of content modification, reason for adaptation and goal). The FRAME organizes the reasons why an adaptation is made into 4 overarching categories: recipient, provider, organization, or sociopolitical context, with specific subcategories. An additional subcategory was developed and added under participant-level reasons for adaptation that emerged from the data—“Health narratives and priorities”—to reflect the adaptations that sought to be more responsive to participants’ understanding and perspectives on their needs and mental health. Descriptive reasons for not implementing the suggested adaptations were further classified into categories inductively developed by the researchers. To organize and streamline findings, the adaptations were clustered by the reason for suggesting them and by whether or not the suggestion was adopted. Strategies for maximizing rigor included progressively reducing the data using a series of defined steps (eg, transcribing, summarizing, charting, and categorizing); using multiple researchers at each step to extract, reduce, and categorize the data; conducting frequent debriefing meetings throughout data collection and analysis; and keeping an audit trail [54,55].

### Ethics Approval

All the procedures were approved by the Institutional Review Boards of the New York State Psychiatric Institute (#7900) and Northwell Health (#20-0429).

## Results

### Overview

A total of 11 CSC clinical providers completed the semistructured interviews: 4 staff members each at 2 sites and 3 staff members at a third site. The interviewed staff members represented different disciplines and clinical roles on the treatment team, including team leaders (3/11, 27%), psychiatric care providers (eg, psychiatrist and nurse practitioner; 5/11, 45%), and primary therapists (eg, clinical social workers; 3/11, 27%). In total, the staff members suggested 31 adaptations (Tables 2 and 3): twenty-four were regarding the intervention itself (eg, app questionnaire and data reports), and the remaining 7 were regarding implementation strategies (eg, reiterating instructions and checking smartphone compatibility). Suggestions to modify content were the most frequent (18/31, 58%) and focused on adding or refining content, such as including new survey questions or displaying additional data in the report. This was followed by suggestions for context modification (13/31, 42%), including aspects of format, such as the design of the report, and aspects of the population, such as which staff should have access to the report. Reasons for suggesting modifications included responsiveness to factors at the participant (15/31, 48%), provider (11/31, 35%), organization or setting (3/31, 10%), and sociopolitical (2/31, 6%) levels. Overall, the goals of the suggested adaptations were to improve the fit with recipients or to increase satisfaction, effectiveness, feasibility, reach, and engagement. Ultimately, 58% (18/31) of suggestions were implemented within the study and applied across all sites (ie, adaptations were not specific to or varied by site), whereas 42% (13/31) were not adopted.

**Table 2:** Summary of adaptations suggested and accepted for mobile health First Episode Digital Monitoring.

Reason for suggested adaptation				Goal was to increase or improve		What was suggested and adapted		Type of adaptation made
**Recipient level**								
	**Health narratives and priorities**							
		Time burden	Fit with recipients, feasibility		Repeat or reassure that skipping some questionnaires is OK		Implementation strategy: content-repeating	
		Privacy or confidentiality	Reach and engagement		Repeat information regarding confidentiality		Implementation strategy: content-repeating	
		Person-centered care	Fit with recipients, satisfaction		Report: use person-centered, experience-based language vs medicalized language		Content: tailoring or tweaking or refining	
		Recovery-oriented approach	Fit with recipients, satisfaction		App: ask how bothersome symptom is and impact on functioning		Content: adding elements	
	**Access to resources**							
		Technology	Reach and engagement, feasibility		Check smartphone compatibility before enrollment		Implementation strategy: context, format	
	**Crisis or emergent circumstance**							
		Participant safety	Fit with recipients		Include suicidal ideation as exclusionary criteria		Context: population	
	**Comorbidities**							
		Multiple mental health symptoms or conditions	Effectiveness		Clarify instructions to include multiple psychiatric medications		Implementation strategy: content-tailoring or tweaking or refining	
		Physical health side effects	Fit with recipients, satisfaction		App: ask about weight gain as potential side effect		Content: adding elements	
**Provider level**								
	**Clinical judgment**							
		Clinically meaningful information	Satisfaction, effectiveness		App: ask about timing of medication use and factor in for adherence		Content: adding elements	
		Clinically meaningful information	Satisfaction, effectiveness		Report: include substance use and caffeine use on report		Content: adding elements	
		Clinically meaningful information	Satisfaction, effectiveness		Report: include lines for daily averages on report’s granular graphs		Content: adding elements	
		Previous training or skills	Feasibility		Train providers to read report and include legend		Training content: adding elements	
	**Preferences**							
		Data visualization	Satisfaction		Report: reduce report or graph density (eg, focus on subset of symptoms or side effects)		Context: format	
		Data visualization	Satisfaction		Report: use dots on report’s granular graphs		Context: format	
**Organization level**								
	**Service structure**							
		Team-based care	Feasibility, effectiveness		Option to share report with multiple staff		Context: personnel	
	**Mission or culture**							
		Shared decision-making	Satisfaction, effectiveness		Option for clinician to show report to the participant		Context: format	
**Sociopolitical level**								
	**Existing policies**							
		COVID-19 pandemic social distancing mandates	Reach and engagement		Attend web-based program meeting for introduction or warm handoff to client for recruitment		Implementation strategy: context, format	
		COVID-19 pandemic social distancing mandates	Reach and engagement		Option to receive an e-gift card as participant reimbursement		Implementation strategy: context, format	

**Table 3:** Summary of adaptations suggested and rejected for mobile health First Episode Digital Monitoring.

Reason for suggested adaptation				Goal was to increase or improve		What was suggested, but not adapted		Type of adaptation not made		Reason why adaptation not made
**Recipient level**										
	**Health narratives and priorities**									
		Time burden	Fit with recipients, feasibility		Reduce ping frequency		Content: shortening or condensing (pacing or timing), tailoring		Compromises core components	
		Time burden	Fit with recipients, feasibility		Tailor ping timing around participant work or school hours		Content: shortening or condensing (pacing or timing), tailoring		Increases complexity	
		Privacy or confidentiality	Reach and engagement		Offer non–app-based means of collecting information		Context: format		Beyond intervention scope	
		Person-centered care	Fit with recipients, satisfaction		App: ask more positively worded questions		Content: adding elements		Increases recipient time burden	
		Person-centered care	Fit with recipients, satisfaction		Ask more open-ended questions		Content: adding elements		Increases complexity of data or report	
	**Access to resources**									
		Technology	Reach and engagement, feasibility		Provide phones to participants		Implementation strategy: content, adding		Additional resources required (as well as in-person meeting during COVID-19 pandemic)	
	**Literacy or education level**									
		Data visualization	Fit with recipients		Report: simplify report so participants can understand it more easily		Content: tailoring or tweaking or refining		Compromises core components (may reduce usefulness to providers as primary targets)	
**Provider level**										
	**Clinical judgment**									
		Clinically meaningful information	Satisfaction, effectiveness		App: ask more about negative symptoms		Content: adding elements		Beyond intervention scope and increases time burden	
		Clinically meaningful information	Satisfaction, effectiveness		App: ask about suicidal ideation		Content: adding elements		Additional resources required	
		Clinically meaningful information	Satisfaction, effectiveness		Allow providers to access more information than what is on the report		Content: adding elements		Additional resources required	
		Clinically meaningful information	Satisfaction, effectiveness		Collect data on days more removed from clinical session		Content: lengthening or extending (pacing or timing)		Beyond scope	
	**Preferences**									
		Data visualization	Satisfaction		Report: use bars on granular graphs		Context: format		Not consistent with most clinicians’ preferences	
**Organization level**										
	**Mission or culture**									
		Shared decision-making	Satisfaction, effectiveness		Send report or information directly to participant		Context: format		Additional resources required and beyond intervention scope	

### Adaptations Suggested and Adopted

#### Adaptations for Participant-Level Reasons

Adaptations that were ultimately adopted were most commonly driven by reasons at the participant level and included the need to address factors such as participants’ health narratives and priorities, comorbidities, access to resources, and safety. With respect to health narratives and priorities, the most substantive changes were to refine or add intervention content. Staff members emphasized the need to use person-centered and experience-based language, instead of medical language, throughout the intervention, including changing data labels on the report (eg, changing “symptoms” to “experiences” and “hallucinations” to “seeing things”; Multimedia Appendix 2):

[It’s important that the] language be recovery-oriented...[many participants] don’t agree with our diagnosis. So that’s why it’s important for us to be able to engage them. It can’t always be- reflect the language of sort of traditional medical model.

P6

Beyond refining the wording, staff members highlighted the need for additional app questions that would incorporate participants’ own perceptions of their mental health in a more person-centered and recovery-oriented way, potentially making the questionnaire more engaging and relevant to the participants. Suggestions that were adopted included adding questions that would not only assess the frequency of symptoms or side effects but also to inquire the degree to which participants perceived these experiences to be bothersome or interfering in their functioning (ie, how much “[This Experience] gets in the way of what I’m doing”):

Particularly for our population, it’s really not about whether or not they have a symptom... It’s really about if that symptom is getting in the way of something...our young people don’t in general tend to like apps that remind them or conceptualize them as being sick... asking things in a way that might be a little more recovery-oriented might be helpful...“If you do experience this, can you tell us...how much is this thing particularly bothersome or impacting your ability to do the things that you want to do whether you work or school”...That way the person could experience it as, “yes I have voices, but no, it’s actually not impacting me” or if something is interrupting your life, it might help you to remind yourself, “okay, this actually is a problem.”

P6

Staff members also noted the need to add questions that would further reflect priorities of the recipients; for example, asking about side effects that were of known concern to them, as subsequently included in the app:

Would you consider adding weight gain to the list of side-effects? Because that’s been something that has been brought up by some participants in the past and the prescriber really tries to... work with them on that.

P3

Staff members also identified the need to reassure participants of confidentiality and voluntariness by repeating content, such as reiterating instructions regarding confidentiality and the ability to skip app questions. Finally, to address concerns regarding participant safety, study exclusionary criteria were modified to include suicidal ideation, whereas concerns regarding the participants’ access to technology were addressed by adding a step to check participants’ smartphone compatibility with the app before enrollment.

#### Adaptations for Provider-Level Reasons

Reasons at the provider level included factors such as clinical judgment, previous training or skills, and provider preferences. Most commonly, this entailed suggestions for adding questions to the app or presenting additional data in the report to maximize access to information that providers believed to be clinically meaningful. This included requests for the report to display specific substances beyond illicit drugs (eg, caffeine) that can impact participants’ functioning and for the app questionnaire to account for different factors that have a role in medication adherence (eg, route of administration and timing):

It doesn’t capture what substance was used. You would have to ask...maybe code each [substance in the report]...show [the participant]...had a cup of coffee...[Caffeine and other substances] are relevant for sleep.

P1

What if the patient’s on a (Long Acting Injectable), like an antipsychotic, how would you capture that?...What if they [were supposed to take] the medication in the morning, but took it in the afternoon...

P11

Suggestions for how best to depict data in the report generally reflected provider preferences for visualizing data in a certain format to enhance readability or to reduce the density of graphs (eg, display only the subset of symptoms and side effects with highest impact or severity) owing to concerns that the report was “a little overwhelming...lots of bars...the page is completely full.” In addition, enhancing training for providers in interpreting reports was identified and incorporated as a key implementation strategy:

At first when I saw the report I’m like, “oh, my gosh, all these dots, all these numbers,” but...you guys [actually] explaining it to me...I feel like it’s really simple...

P3

#### Adaptations for Organization-Level Reasons

Regarding reasons associated with the organization or setting, adaptations were suggested to better align the project with key aspects of the mission or culture of the CSC programs and team service structure, specifically SDM and the use of a team-based approach. For example, staff members suggested that they could show the report to participants during sessions, using it as a “visual” tool for promoting participant engagement and informing SDM processes (eg, discussing options, tailoring pros and cons, and exploring patient fears or expectations):

I could totally see using it. I’m all about transparency. So I would show [the report] to them, and I would try to explain it and everything. “And this is what the data says...” in terms of engaging them into their treatment, it’ll help with that...this is...shared decision making. And this gives them more of a connection and participation in their treatment.

P1

Furthermore, given the multidisciplinary and team-based approach of the CSC programs, providers emphasized that team members other than the psychiatric care provider should have access to the report, which was integrated as an option:

Since we are a team and we talk very openly about each participant...I think all of our team members should get [the report]...it would be like a comprehensive way to say...this person is...experiencing this and this, experiencing this kind of side-effects, and then we can get together as a team about it during our meeting.

P8

#### Adaptations for Sociopolitical Context–Level Reasons

Finally, to respond to the sociopolitical context, adaptations to implementation strategies were suggested to address some of the barriers related to COVID-19 pandemic social distancing mandates. Given the limited in-person services, staff members noted the need to expand options for reimbursing participants (eg, offering electronic gift cards) and for preserving aspects of a warm handoff when linking participants to researchers by adding the option of a web-based handoff:

To introduce the [participant]...we are able to do groups via the [virtual] platform. So if the participant is able to go onto the platform and do our video session...if they agree, [the research assistant] can join and it will be the three of us.

P8

### Adaptations Suggested but Not Adopted

Of the 31 suggestions, 13 (48%) adaptations were ultimately not implemented, which generally reflected suggestions to add content by collecting additional information through the app questionnaire, to adapt aspects of context to facilitate participants’ direct access to and understanding of their own data, and to change the pacing or timing of the intervention components. Overall, the reasons for suggesting these adaptations reflected rationales similar to those behind the adaptations that were made, with responsiveness to health narratives and priorities of the participants and clinical judgment of the providers once again being the most frequent. The reasons that researchers did not incorporate these suggested adaptations included additional study resources being required, modifications being beyond the scope of the intervention, concerns regarding compromising core components or mechanisms, managing intervention complexity, managing participant time burden, and adaptations not being consistent with the preferences of most providers. The staff suggested additional questionnaire content, such as asking more about negative symptoms and positive experiences or adding open-ended questions, primarily as a potential way to make the app more engaging for participants:

But it will also be nice to, towards the end, to say oh, “but you did report this other positive thing that happened to you.” Or so it’s just not about medication.

P10

Although researchers acknowledged the potential value of collecting this additional data, these additions were ultimately not made owing to concerns that they were outside the primary scope of the pilot trial, would pose an increased time burden for participants, or would unacceptably increase the complexity of the data presented in the report.

The staff also expressed concerns about different aspects of intervention timing, inquiring “how realistic” it was for participants to respond to 10 questionnaires a day, with suggestions to reduce or tailor questionnaire frequency. There was also provider uncertainty about the timing of data collection, with suggestions to space out participant completion of questionnaires and to include time points further removed from upcoming appointments to potentially capture experiences that may also be relevant but more challenging to remember:

Is there an opportunity to have flexibility with what three days are selected...As opposed to the last three days before they’re seeing me...answering those questions [at different points] in real time further away from my appointment...I could see sometimes where [the past three days] might matter, if there’s something they want to talk about in their experience more recently. I can see sometimes where it’s not as relevant.

P6

These changes to intervention timing were not adopted, with researchers seeking to preserve the core component of 10 ESM questionnaires based on their prior experience of high frequencies yielding adequate response rates [13], and tailoring questionnaire frequency to participants’ changing schedules was too complex to be reliably implemented over time.

Providers also suggested offering alternative means for participants to complete the questionnaire, offering smartphones to participants lacking the technology, as well as providing participants with direct access to their own data and further simplifying the report to make it easier for participants to understand:

Is there an option if participants are hesitant about downloading an app, like a way to do it by email...

P2

It would be nice if when you’re with a particular client to simplify these graphs. Because if you are going to use it as a tool, like this most people would not understand.

P10

Although these suggestions had the potential to expand intervention reach and enhance participant engagement with the intervention and their own data, they were ultimately not adopted. Researchers determined that offering a non–app-based means of collecting data was beyond the scope of the mHealth intervention and that tailoring the report to participants versus providers could result in a loss of information that potentially compromised core components. Moreover, purchasing smartphones would require additional funding.

## Discussion

### Principal Findings

This study presents our process and findings of using rapid and pragmatic qualitative methods along with the FRAME to systematically solicit, document, deliberate, and report provider-suggested adaptations to FREEDoM, an mHealth app aimed at enhancing treatment for individuals with FEP. This study is one of only a handful of published reports characterizing efforts to incorporate direct stakeholder input (eg, clinicians) into the development process of an app targeting treatment of psychosis and the first to focus on enhancing the therapeutic relationship and improving SDM among patients with FEP and their treatment teams.

With overarching research questions guided by the FRAME, we conducted focused semistructured interviews while concurrently extracting data from transcripts to interview summaries and then to a descriptive matrix, further condensing the data at each step until we categorized each adaptation along the FRAME domains. This study demonstrates how these methods can facilitate rapid analysis of qualitative research data for intervention adaptation and yield timely findings with high clinical relevance to inform the delivery of care.

Reasons for suggesting adaptations most commonly included responsiveness to health narratives and priorities of patients, clinical judgment of providers, and mission or culture of organizations. Suggestions to add or refine content were most common, including asking participants to rate how bothersome symptoms or side effects were, rewording the report to be person centered and experience based in lieu of medical language, and presenting additional data in the report. Adaptations to context were most often related to an implementation strategy (eg, web-based handoffs during recruitment), the format of the provider report, and with whom the report was shared.

Overall, the adaptations that were suggested and adopted were driven by key aspects of the CSC context to shift the intervention to better reflect the needs and preferences of the population served and the CSC’s emphasis on SDM, recovery-oriented practice, and team-based approach to care. In particular, asking additional questions and changing the phrasing of report labels sought to address factors such as patients’ perceptions of their mental health conditions, priorities, and existing comorbidities. The inclusion of additional questions also addressed providers’ need for more comprehensive and clinically relevant information, as did changes to which data were displayed and how the report was designed. Adaptations implemented also responded to key aspects of the structure, mission, and culture of the CSC programs. For example, the CSC team-based approach to care necessitated the option of sharing the report across providers, whereas the option to review the report collaboratively with participants during a session aligned with SDM. This adaptation to share the report with other providers and patients, as well as the inclusion of patients’ perceptions of the impact of symptoms on functioning, may be particularly important to counteract the potential tendency of any one provider to narrowly interpret or selectively focus on certain data, given their particular role, background, or training. Although not fully eliminating factors such as providers’ information selection bias, incorporating patients’ ratings of functioning and having multiple individuals review and discuss the report, including the patients themselves, may help bridge the gap between what patients and any one provider might perceive as important, relevant, or possible, potentially enhancing SDM.

Adaptations that were suggested but not incorporated most frequently reflected suggestions to collect additional patient information, facilitate patients’ access to their own data, or change the timing of the intervention components. The fact that the rationales for suggesting these adaptations, which were ultimately not made, were generally similar to the those for implemented adaptations indicates that the adaptation decision-making process—whether to adapt or not—did not appear to exhibit a systematic bias (eg, consistently rejecting adaptations reflecting participant-level compared with provider-level factors). Suggested adaptations were not incorporated into the intervention when the research team deemed that they were outside of the current aims or scope of the trial, potentially compromised core components or mechanisms or that they presented a feasibility challenge such as insufficient resources to implement an adaptation in the context of a pilot trial or increased complexity.

The tracking of adaptations not made further helps to highlight key dilemmas that may frequently emerge when deliberating mHealth adaptations within clinical care. For example, in this study, researchers had to weigh the potential benefit of the providers’ suggestion that participant engagement could be encouraged by including more positively worded statements or open-ended questions in the app against the potential drawback of increased time required to complete questionnaires, which might discourage participant engagement. Ultimately, the decision was made to not include these extra questions, given that the potential net impact on engagement was unclear. In addition, it hindered the study’s ability to expeditiously produce short 1-page clinician reports by having to process and include additional items and free text entries, which would also potentially increase the amount of time that clinicians would need to review a more complex report. Such deliberations illustrate how decision-makers may have to discern how best to balance factors such as the desire to potentially create a more engaging app while not sacrificing feasibility by inadvertently creating an excessive time burden for patients or clinicians. Future studies can further identify the information that decision-makers consider when weighing these factors and explore the feasibility of empirically pretesting different iterations of an intervention when the evidence to support an adaptation decision is unclear. For example, with adequate time and resources, 2 versions of an app could be tested—one with and one without the positive and open-ended questions—providing an empirical basis upon which to accept or reject this suggestion, depending on the respective rates of participant engagement. Overall, tracking adaptations not made provides greater insight into the dilemmas and decision-making processes of intervention adaptation while also offering concrete suggestions that can be considered for future refinement of similar mHealth interventions. Proposing preliminary categories for reasons why adaptations are not made represented the first step toward providing guidelines to standardize this process.

Overall, health care systems and workflows often vary dramatically, necessitating consideration of whether to integrate uniform and standardized interventions or shape interventions around specific aspects of local contexts, for example, the needs, preferences, and training backgrounds of providers in any setting. Adapting interventions to certain contexts and providers may yield several benefits, such as increased intervention uptake, satisfaction, and effectiveness. However, the challenges in engaging in the process of intervention adaptation include the extra time, resources, and expertise required to solicit stakeholder input and make adaptations. By illustrating some of the tools and rapid approaches used in this study, we seek to help minimize some of these challenges.

### Limitations

This study has several limitations. Although CSC providers with different clinical roles were interviewed, the inclusion of other provider roles representing nonclinical staff (eg, peer specialist and supported employment specialist) could have yielded additional information relevant to adaptation, particularly given the team-based approach of CSC programs. The inclusion of CSC patients was originally planned as part of stakeholder interviews (to be published in a separate manuscript); however, the onset of the COVID-19 pandemic and the enactment of social distancing mandates coincided with the start of the study and interfered with patient data collection. Given that implementation barriers identified by patients and providers can be different, the inclusion of CSC patients would likely have identified additional suggestions that either expanded upon or potentially conflicted with the feedback offered by providers. Future studies, including our pilot trial of the developed FREEDoM app, which includes both stakeholder perspectives, can also offer insights into how best to balance or reconcile suggested adaptations that differ or conflict between patients and providers. Nevertheless, by soliciting CSC clinicians’ perspectives, this study addressed a key gap in the literature regarding providers’ information needs and strategies that may promote mHealth integration into early psychosis treatment. This gap is particularly important to address given the overarching concerns regarding providers’ buy-in for, and use of, patient-generated data in health care more broadly [40]. In addition, although our study contributes to the current understanding of provider preferences regarding MBC within early psychosis treatment and how to deploy mHealth technologies, it represents only an initial step, with much work remaining to identify the factors that influence long-term implementation, acceptability, and sustainability.

By virtue of the research objective, identified adaptations reflect the context of participating CSC programs and the scope of a subsequent clinical trial seeking to provide clinicians with patient information that may impact pharmacological treatment decisions. However, CSC is an established evidence-based practice with well-articulated core components that may support broader applicability of our findings, including a team-based approach, a wide range of multidisciplinary services (eg, psychotherapy, pharmacotherapy, and primary care coordination; supported employment and education; family education and support; and case management), and person-centered, recovery-oriented treatment that emphasizes SDM. In addition, although all 3 CSC study sites were in urban areas and had their fidelity to the model monitored, adaptations were uniform across sites despite variability along other key dimensions, such as the type of organization operating the program (eg, affiliated with a community-based nonprofit organization vs a hospital), aspects of population served (eg, ratio of more newly enrolled CSC patients to more established patients), and psychiatric or medical staffing (eg, nurse practitioner or psychiatrist, one or multiple psychiatric providers on team). Although this suggests the potential for broader generalizability of findings across CSCs, the adaptations may not be applicable for settings using mHealth data for a different purpose or to CSC programs that substantially depart from the model’s core functions and components, particularly those that may not adopt the recovery-oriented, person-centered, and SDM approaches that drove many of the adaptations suggested in this study. Finally, the study focused on adaptations suggested before intervention implementation; therefore, results from ongoing clinical trials are needed to evaluate the implementation and effectiveness of the developed mHealth intervention.

### Conclusions

This study illustrates a pragmatic and rapid application of the FRAME to track provider-suggested adaptations to FREEDoM, a novel mHealth intervention app, and its implementation within “real-world” FEP treatment programs. The methodology used in this study offers a rigorous, iterative, and rapid approach to solicit, analyze, and incorporate qualitative stakeholder inputs for the development and adaptation of clinical interventions. Systematic tracking of suggested adaptations, including which adaptations were ultimately not implemented (and why), is essential to understanding and enhancing key implementation indicators such as intervention fit, feasibility, and acceptability while also increasing transparency and accountability in the adaptation decision-making processes. The FREEDoM app seeks to enhance the therapeutic relationship and improve SDM between patients with FEP and their treatment teams. Future studies should characterize relevant clinical findings, including measures of therapeutic relationships and SDM.

## Appendix

Multimedia Appendix 1 Clinician qualitative interview.

Multimedia Appendix 2 Sample participant mobile health reports.

## Abbreviations

CSC: coordinated specialty care
ESM: experience sampling method
FEP: first-episode psychosis
FRAME: Framework for Reporting Adaptations and Modifications‐Enhanced
FREEDoM: First Episode Digital Monitoring
MBC: measurement-based care
mHealth: mobile health
RCT: randomized controlled trial
SDM: shared decision-making
