# Supplementary material for: Development of the First Episode Digital Monitoring mHealth Intervention for People With Early Psychosis: Qualitative Interview Study With Clinicians
Source: JMIR Ment Health. 2022 Nov 4;9(11):e41482. doi: 10.2196/41482 (PMC9675009; doi:10.2196/41482)
Supplement: Multimedia Appendix 1 [file mental_v9i11e41482_app1.pdf]

mHealth for Optimizing Pharmacotherapy
Stakeholder: OnTrackNY Personnel (e.g., team leader, prescriber, primary clinician, peer, program director)

Objectives:
The goal of these interviews is to reveal potential implementation strategies and challenges which will inform adaptations of the mHealth intervention. Specifically, the aims are:

1. To understand each OnTrackNY site's current practices regarding medications, including communications between clinicians, visit scheduling, and clinic logistics practices for individuals with schizophrenia.

2. To obtain stakeholders' feedback on the mHealth application prototype and mock reports of patient-reported information.

Interviews will last about 45 minutes and will be audio-recorded and transcribed.

Introduction:
During this interview, we'll ask you questions about the clinic's practices around medication prescribing for OnTrackNY participants. We will be using a smartphone application to get more detailed information from patients about symptoms, side effects, daily activities that we think will be useful to help optimize a person's medications. We also want your feedback on the app, the way to report the information, and how to implement the study at your site.

Current Role:
We'll start with some questions about your role with OnTrackNY and how participant visits are scheduled at this clinic.

1. What is your current role at OnTrackNY?

2. What role do you have in participant care?

3. What challenges do you see with medications in your participants? Adherence? Side effects? Persistent symptoms?

Overview of the mHealth intervention:
This next section of the interview will ask about your overall impressions of the mHealth intervention.

[App demonstration]

Overall, the goal of our program is to improve prescription choices by asking participants about their symptoms and side-effects as they experience them, rather than

relying on them to keep track of these things between appointments. Using the app I just demonstrated, people in the program will answer questions about their symptoms and side-effects 10 times per day for 3 consecutive days every 2 weeks. The questionnaires are short, about 3-4 minutes each. We will use the answers to prepare a summary report will be given to each participant's team and prescriber to help make decisions about medications.

4. Do you have any overall questions about what we plan to do?

5. What do you think about the app?
    a. What do you like best?
    b. What do you like least?

6. How would this program fit into your overall care for patients?

7. What challenges might arise from using this program?
    a. How might it interfere with your overall care for patients?

8. What benefits might this program provide?
    c. How could this benefit patients?
    d. How could this improve the overall care you provide to patients?

Feedback on Report:
During a patient's participation in this study, you will be provided with reports on the data we collect from them.

[Display report(s)]

9. What do you think about the draft report?
    a. What do you like best?
    b. What do you think of how the information is organized?
    c. What needs to be changed?

10. What would make this report better or more useful?
    d. What information is missing? What else would you like to know?
    e. What information is most critical for patient care?

11. Who should see these reports? When? How often?

12. We are considering making additional patient information available to you online. Do you think that would be useful?

13. What difficulties or challenges do you see with using the mobile health app and reports?
    - Challenges for you?
    - Challenges for patients/participants?

- What could help address these challenges?

14. How should we go about implementing the mHealth intervention at your organization?
    a. What resources may be needed in terms of training and infrastructure?

Overall Impressions:
I'd like to ask a few final questions about the study's logistics.

15. What do you think would be the most efficient strategy to recruit participants into the study?

16. What factors might make it challenging to recruit participants in this study? How might these be addressed?

17. Once potential participants are enrolled, what can be done to keep participants engaged in the study (i.e., completing study assessments)?

18. Some people will get randomized to the control condition. What type of support would participants who do not get the mHealth intervention need to stay engaged with the study?

19. After everything we have discussed, what do you think are the most important factors to help us implement this mHealth study to make it successful?
