# Supplementary material for: Development of the First Episode Digital Monitoring mHealth Intervention for People With Early Psychosis: Qualitative Interview Study With Clinicians
Source: JMIR Ment Health. 2022 Nov 4;9(11):e41482. doi: 10.2196/41482 (PMC9675009; doi:10.2196/41482)
Supplement: Multimedia Appendix 2 [file mental_v9i11e41482_app2.docx]

Sample Reports

Pre-Adaptation mHealth FREEDoM Draft Report


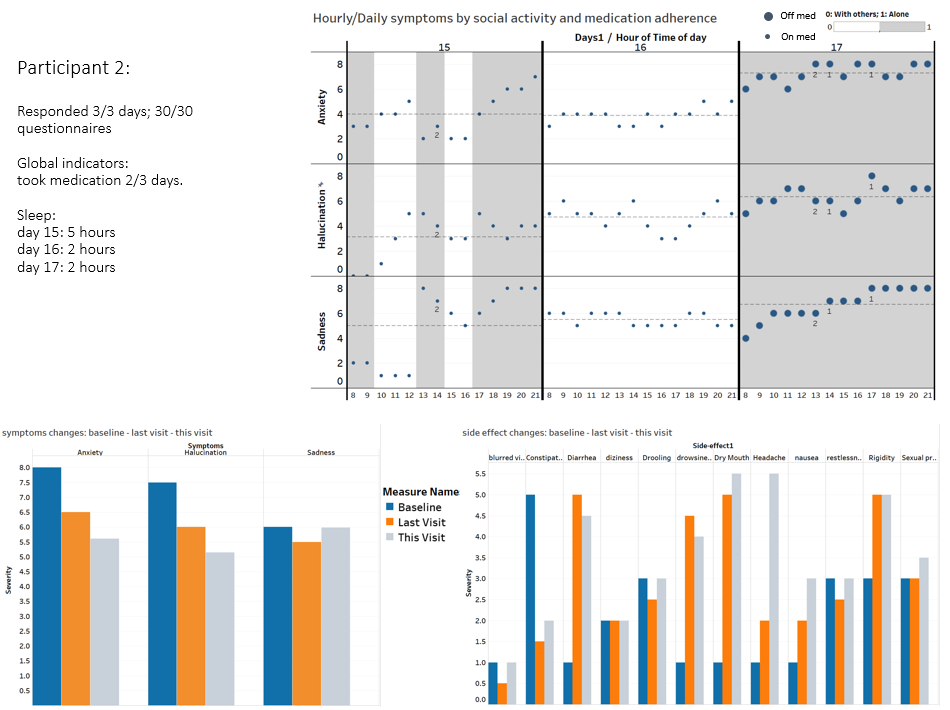


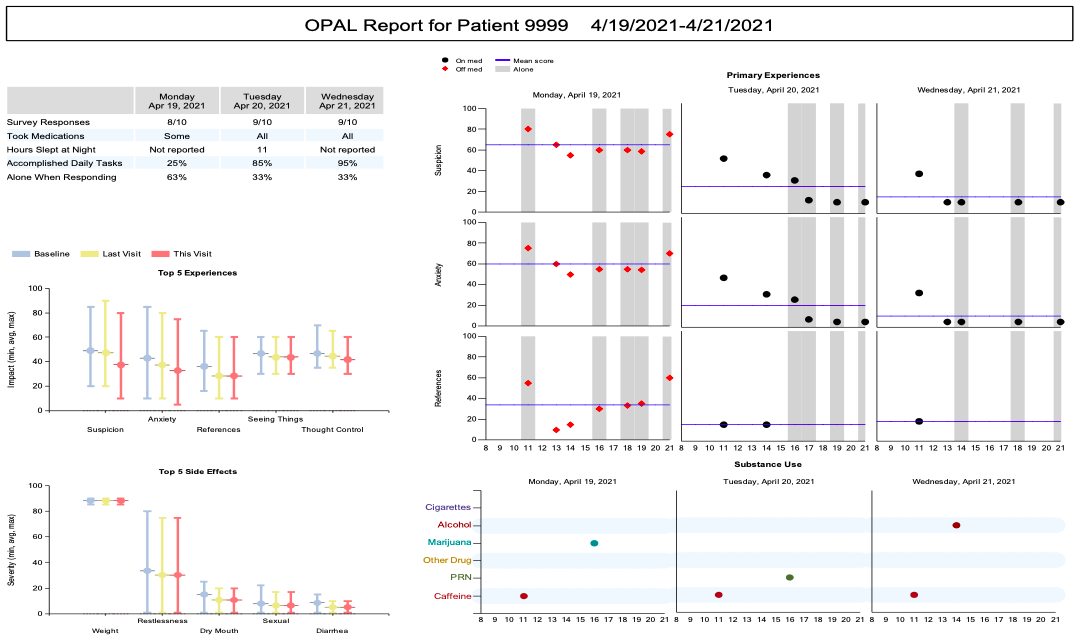
 Post-Adaptation mHealth Freedom Sample Report
